# Intelligence

**Published:** 1833-06

**Authors:** 


### INTELLIGENCE.

### KING'S COLLEGE, LONDON.

On Saturday last, the 25th ultimo, the prizes awarded to the successful candidates in the several classes of the medical department were distributed, at a public meeting held in the large theatre, in the College. The Bishop of London presided on the occasion.

The following is a list of the students to whom prizes and certificates of honour were adjudged.

| | | |
|---|---|---|
| First gold medal, for general medical proficiency | | Mr. T. E. Rawson. |
| Second ditto | | — E. J. Chance. |
| Anatomy | Silver medal | Mr. W. T. C. Robinson. |
| | First certificate, | — J. Soden. |
| | Second ditto | — A. H. Talmadge. |
| | Third ditto | — Galland. |
| Anatomical Demonstrations | Silver medal | Mr. John Soden. |
| | Certificate | — W. T. C. Robinson. |

| | | |
|---|---|---|
| BOTANY ............................ | Silver medal..... | Mr. W. B. Whitfield. |
| | First certificate, | — E. J. Chance. |
| | Second ditto...... | — H. C. Medcalfe. |
| CHEMISTRY........................ | Silver medal... . | Mr. James Orwill. |
| | First certificate, | — H. Curling. |
| | Second ditto ...... | — T. E. Rawson. |
| | Third ditto ..... | — H. C. Medcalfe. |
| MATERIA MEDICA .................. | Silver medal..... | Mr. H. Holme. |
| | First certificate, | — T. E. Rawson. |
| | Second ditto..... | — J. F. Osborn. |
| MEDICINE ........................ | Silver medal..... | Mr. H. C. Medcalfe. |
| | First certificate, | — C. T. Carter. |
| | Second ditto...... | — T. E. Rawson. |
| | Third ditto .. ... | — G. Winchester. |
| FORENSIC MEDICINE .............. | Certificate ...... | Mr. E. J. Chance. |
| MIDWIFERY.......................... | Silver medal...... | Mr. H. C. Medcalfe. |
| | First certificate, | — T. E. Rawson. |
| | Second ditto.. ... | — G. Pearse. |
| SURGERY ........................... | Silver medal...... | Mr. W. Trew. |
| | First certificate, | — C. T. Carter. |
| | Second ditto.. ... | — T. E. Rawson. |
| | Third ditto ...... | — A. H. Talmadge. |

Mr. LEATHE's prizes for regularity of conduct, and attention to religious duties ......... ..... { Mr. Leacock. — Atkinson. }

### MONTHLY LIST OF MEDICAL BOOKS.

### METEOROLOGICAL REGISTER,

By Messrs. HARRIS and Co., Mathematical Instrument Makers, 50, High Holborn.

| | Moon. | Rain gauge. | Thermometer | | | Barometer. | | De Luc's Hygrometer | | Winds. | | Atmospheric Variation. | | |
|---|---|---|---|---|---|---|---|---|---|---|---|---|---|---|
| Apr. | | | 9 a.m. | max. | min. | 9a.m. | 10p.m. | 9a.m. | 10p.m. | 9 a.m. | 10p.m. | 9 a.m. | 2 p.m. | 10 p.m. |
| 20 | ● | | 48 | 55 | 46 | 29.88 | 29.90 | 73 | 68 | W | NNW | rain | fine | fine |
| 21 | | | 52 | 56 | 44 | .94 | .94 | 64 | 69 | E | SW | fine | | |
| 22 | | | 48 | 59 | 38 | .99 | .98 | 69 | 69 | W | N | foggy | | |
| 23 | | | 42 | 47 | 48 | .98 | .94 | 69 | 70 | N | W | | | cloudy |
| 24 | | | 55 | 59 | 43 | .78 | .85 | 72 | 72 | ESE | ENE | cloudy | | cloudy |
| 25 | | .10 | 51 | 57 | 43 | 30.02 | 30.04 | 73 | 73 | NE | NE | cloudy | cloudy | fine |
| 26 | | | 49 | 60 | 52 | .02 | 29.94 | 73 | 72 | NNW | NW | fine | fine | fine |
| 27 | ☽ | | 56 | 62 | 47 | 29.82 | .66 | 72 | 70 | WNW | NW | showery | | |
| 28 | | | 51 | 58 | 43 | .58 | .47 | 69 | 68 | WNW | W | cloudy | | |
| 29 | | | 52 | 55 | 43 | .40 | .39 | 68 | 68 | WSW | W | cloudy | | |
| 30 | | | 53 | 59 | 45 | .42 | .48 | 68 | 67 | WNW | NW | | | |
| May 1 | | | 55 | 60 | 52 | .46 | .40 | 67 | 72 | WSW | WNW | fine | | cloudy |
| 2 | | | 55 | 59 | 54 | .30 | .48 | 75 | 77 | SW | SSW | showery | rain | fine |
| 3 | | | 54 | 61 | 57 | .60 | .65 | 81 | 79 | SW | WSW | overcast | fine | |
| 4 | ○ | | 62 | 68 | 58 | .70 | .80 | 75 | 76 | WSW | NNW | fine | | |
| 5 | | | 65 | 70 | 52 | .97 | 30.19 | 65 | 63 | NE | SE | | | |
| 6 | | | 63 | 66 | 50 | 30.24 | .24 | 63 | 61 | ESW | SSE | | | |
| 7 | | | 61 | 65 | 52 | .16 | .01 | 60 | 62 | SE | E | | | cloudy |
| 8 | | | 61 | 67 | 53 | 29.86 | 29.75 | 65 | 65 | ESE | SE | cloudy | | fine |
| 9 | | | 64 | 73 | 53 | .60 | .57 | 65 | 65 | S | WSW | fine | | |
| 10 | | | 62 | 65 | 54 | .75 | .80 | 65 | 65 | WSW | WSW | cloudy | | |
| 11 | ☾ | | 61 | 67 | 57 | .87 | .90 | 65 | 64 | WSW | SE | cloudy | | |
| 12 | | | 65 | 73 | 55 | .85 | .79 | 64 | 64 | SW | SW | fine | | |
| 13 | | | 62 | 71 | 55 | .83 | .89 | 64 | 64 | NW | WNW | | | |
| 14 | | | 64 | 70 | 59 | .97 | .88 | 64 | 63 | W | SSW | | | cloudy |
| 15 | | | 75 | 80 | 65 | .77 | .70 | 62 | 58 | S | S | | | fine |
| 16 | | | 75 | 78 | 62 | .70 | .72 | 55 | 55 | SW | SW | | | |
| 17 | | | 76 | 79 | 61 | .64 | .81 | 56 | 56 | SE | NNW | | | |
| 18 | | | 62 | 68 | 54 | 30.02 | 30.07 | 56 | 55 | NE | SE | cloudy | | cloudy |
| 19 | ● | | 58 | 62 | 54 | 29.94 | 29.96 | 59 | 75 | NE | E | fine | rain | |

The quantity of Rain fallen in April, was 1 inch and 22.100ths.

